# Reply to López-Moreno, M. Comment on “Montoro-García et al. Beneficial Impact of Pork Dry-Cured Ham Consumption on Blood Pressure and Cardiometabolic Markers in Individuals with Cardiovascular Risk. *Nutrients* 2022, *14*, 298”

**DOI:** 10.3390/nu14204282

**Published:** 2022-10-14

**Authors:** Silvia Montoro-García, Ángeles Velasco-Soria, Carmen Carazo-Díaz, Fidel Toldrá, Antonio Avellaneda, José Abellán-Alemán

**Affiliations:** 1 Department for Cardiovascular Risk, Faculty of Health Sciences, UCAM Catholic University of Murcia, Campus los Jerónimos, 30107 Murcia, Spain; 2 Cátedra de Estadística “Big Data”, Campus los Jerónimos, UCAM Catholic University of Murcia, 30107 Murcia, Spain; 3 Instituto de Agroquímica y Tecnología de Alimentos, CSIC, Av. Agustín Escardino 7, 46980 Valencia, Spain; 4 R&D Department, ElPozo Alimentación S.A., 30840 Alhama de Murcia, Spain

We thank Dr. López -Moreno for the comment [1] on our publication [2]. Traditionally, the inclusion of dry-cured pork ham in the diet of patients with cardiovascular risk and, more particularly, in hypertensive patients is contraindicated due to its high salt content. However, the in vitro and in vivo inhibitory effects exerted by certain bioactive peptides that are generated during the curing process, mainly on the angiotensin-converting enzyme (ACE), led to suspect effects of lowering blood pressure that were against the pressor effect derived from the higher salt intake that this meat consumption implies.

We obviously agree that the consumption of fruits, vegetables, as well as foods rich in potassium and active compounds display well-known cardiovascular benefits. Nonetheless, the objective of this study was not to compare the effect that regular consumption of dry-cured pork ham exerts in the primary prevention of cardiovascular diseases to the consumption of a diet rich in vegetables. 

On the contrary, the objective of this study was to confirm the potential role of bioactive peptides, included in dry-cured ham, on blood pressure and other metabolic parameters. For this, the clinical study has to be designed with another meat product where we would make sure that such peptides were absent but displayed a similar level of salt concentration, such as cooked ham.

Table 1 shows that, in our study, after consuming cooked ham—high salt content but no bioactive peptides—the means of all blood pressure parameters tend to increase. In two of those parameters (night diastolic BP and % high SBP) this increase was even statistically significant. However, the trend of the clinical variables after consuming dry-cured ham—high salt content but bioactive peptides—was to reduce these variables, being the reduction in % high SBP statistically significant (Table 1). Additionally, we were also able to detect a trend to decrease blood glucose and serum cholesterol levels, as well as other metabolic markers. All of this despite the intake of 1.5 g/day of salt higher than the control product. This obviously does not directly confirm that such effect is due to the bioactive peptides but at least provide evidence to eliminate the contraindication of moderate consumption of dry-cured pork ham in patients with cardiovascular risk and certainly encourages a larger study including vegetables and fruits in both branches to test whether these results are confirmed in a more suitable diet. 

Regarding the conflict of interest, we must reaffirm that there is no such conflict since the meat products used had to be obtained from somewhere. In our study, it was produced in EL POZO ALIMENTACION, which under the direction of Instituto de Agroquímica y Tecnología de Alimentos (IATA) researchers, obtained a product cured with a concentration of low salt, in which 13 biopeptides with the capacity to inhibit ACE were detected. Authors from ELPOZO did not contribute to the design of the clinical study and never had access to the results of the study.

## Figures and Tables

**Table 1:** Mean effect and 95% confidence interval after eating dry-cured ham and cooked ham.

Variable	Dry-Cured Pork Ham Mean and IC al 95%	Cooked Ham Mean and IC al 95%
BMI, (kg/m^2^)	−0.09 (−0.27 and 0.09)	0.08 (−0.07 and 0.23)
Fat content, %	−0.40 (−0.91 and 0.11)	−0.07 (−0.59 and 0.44)
Mean 24 h Systolic BP	−1.62 (−3.58 and 0.34)	0.65 (−0.71 and 2.00)
Day Systolic BP	−1.58 (−3.72 and 0.56)	0.06 (−1.54 and 1.66)
Night Systolic BP	0.02 (−2.27 and 2.31)	2.00 (0.19 and 3.81)
Mean 24 h-Diastolic BP	−1.20 (−2.72 and 0.32)	1.08 (−0.09 and 2.26)
Day Diastolic BP	−1.06 (−3.36 and 1.24)	0.81 (−0.91 and 2.53)
Night Diastolic BP	−0.41 (−4.06 and 3.24)	0.57 (−2.11 and 3.26)
MAP	−1.34 (−2.92 and 0.24)	0.94 (−0.17 and 2.05)
% High SBP	−2.73 (−5.36 and −0.09)	2.61 (0.16 and 5.06)

BMI: Body Mass Index; BP: Blood Pressure; MAP: Mean Arterial Pressure; SBP: Systolic Blood Pressure.
